# Green Light Partial Replacement of Red and Blue Light Improved Drought Tolerance by Regulating Water Use Efficiency in Cucumber Seedlings

**DOI:** 10.3389/fpls.2022.878932

**Published:** 2022-05-31

**Authors:** Yuting Ma, Linli Hu, Yue Wu, Zhongqi Tang, Xuemei Xiao, Jian Lyu, Jianming Xie, Jihua Yu

**Affiliations:** ^1^College of Horticulture, Gansu Agricultural University, Lanzhou, China; ^2^Basic Experiment Teaching Center, Gansu Agricultural University, Lanzhou, China; ^3^Gansu Provincial Key Laboratory of Aridland Crop Science, Gansu Agricultural University, Lanzhou, China

**Keywords:** cucumber (*Cucumis sativus* L.), green light, drought stress, water use efficiency, GABA

## Abstract

Light is one of the most important environmental signals in plant growth, development, and stress response. Green light has been proved to enhance plant defense against biotic and/or abiotic stress. To illustrate the effects of green light partially replaced red light and blue light on the plant under drought condition, cucumber (*Cucumis sativus* L. cv. Xinchun No. 4) seedlings were treated with short-term drought stress and were concomitantly exposed to four treatments, which were set up by adjusting the relative amount of green light as 0 (RB), 25 (RBG25), 50 (RBG50), and 75 (RBG75) μmol m^−2^ s^−1^, respectively, with a total photosynthetic photon flux density of 250 μmol m^−2^ s^−1^ and a fixed red-to-blue ratio of 4:1. The results showed that compared with RB, RBG50 significantly increased shoot fresh weight (FW) and dry weight (DW), root DW, plant height, stem diameter, leaf area, and leaf dry mass per unit area (LMA) by 10.61, 7.69, 66.13, 6.22, 10.02, 4.10, and 12.41%, respectively. Also, the addition of green light significantly increased the root volume and root tip number. Moreover, green light partial replacement of red light and blue light increased total water content, especially free water content, improved leaf water status, and alleviated water loss in plants caused by drought stress. Also, the addition of green light increased net photosynthetic rate (P_n_), reduced both stomata conductance (g_s_) and transpiration rate (E), enhanced the intrinsic water-use efficiency (WUE) and instantaneous water-use efficiency (iWUE) of leaves, and increased the content of chlorophylls a and b. Green light substituting a proportion of blue and red light regulated stomatal aperture by significantly increasing abscisic acid (ABA) and γ-aminobutyric acid (GABA) content. In addition, the increase of GABA was resulted from the upregulation of *Glutamate Decarboxylase 2* (*CsGAD2*). However, the relative electrolytic leakage and contents of malondialdehyde (MDA), superoxide anion (${\text{O}}_{2}^{-}$), and hydrogen peroxide (H_2_O_2_) vigorously decreased as the intensity of green light was added to the spectrum under drought. Conclusively, green light partially replaced red light and blue light and improved drought tolerance of cucumber seedlings by upregulating the expression of *CsGAD2* gene and promoting the synthesis of GABA. The increase in GABA content further downregulated the expression of *aluminum-activated malate transporter 9* (*CsALMT9*) gene, induced stomata to close, improved water utilization, and alleviated damage caused by drought. This study highlights a role of green light in plant physiological processes. Moreover, analyzing the function of green light on improving drought tolerance of plants could open alternative avenues for improving plant stress resilience.

## Introduction

Drought wreaks havoc on ecosystems, severely affects the plant growth, and reduces food production (Gallagher et al., 1976; Witze, 2014). It is reported that 70% of global freshwater is used for agriculture (Döll, 2009). Stomatal regulation is one of the key determinants of plant productivity and drought tolerance, and its impact on the global carbon and water cycles can further influence the climate (Hetherington and Woodward, 2003; Keenan et al., 2013; Papanatsiou et al., 2019). Stomata can be regarded as hydraulically driven valves in the leaf surface, which open to allow CO_2_ uptake for photosynthesis while simultaneously close to prevent excessive loss of water, essential for plant survival in dry conditions (Macrobbie, 2006; Kim et al., 2010; Papanatsiou et al., 2019). Chloride, malate, and nitrate are considered to be the main anions involved in the stomatal movement (Hedrich, 2005; Kim et al., 2010; Barbier-Brygoo et al., 2011). Large and fast changes in ion concentration are the important ways to regulate stomatal closure (Macrobbie, 2006), which are often mediated by ion channels (MacRobbie, 1998; Roux and Leonhardt, 2018). Among the anion transporter or channel families identified so far, aluminum-activated malate transporters (ALMTs) form a unique family of passive transport systems that are exclusive to plants (Kovermann et al., 2010; Meyer et al., 2010; Sasaki et al., 2010; De Angeli et al., 2013; Cornelia et al., 2017; Ramesh et al., 2018). Stomatal guard cells contain a number of ALMTs that impact stomatal movement and transpirational water loss (Meyer et al., 2010; De Angeli et al., 2013; Cornelia et al., 2017). ABA induces stomatal closure through ALMT4 (Cornelia et al., 2017). GABA is a regulated signal of ALMT9 activity (Ramesh et al., 2018; Bo et al., 2021; Xu et al., 2021). Stomata are very important for plants to respond to stress tolerance, including stomatal shape, size, and stomatal aperture. Compared with kidney stomata, plants with dumbbell-shaped stomata can respond quickly and efficiently to changing light conditions and improving their chances of avoiding drought (Hetherington and Woodward, 2003). Moreover, Retallack and Gregory (2001) found a significant negative correlation between stomatal length and drought sensitivity in six tree species. In these species, large stomata close more slowly and are more likely to develop hydraulic dysfunction under drought conditions. Small stomata open and close more quickly, and their general association with high density provides the ability to increase stomatal conductance rapidly in leaves, maximizing the diffusion of CO_2_ into leaves under conditions conducive to photosynthesis (Retallack and Gregory, 2001). Moreover, plants can regulate drought tolerance and salt tolerance through stomatal aperture (Huang et al., 2009).

Movement of stomata is regulated by environmental conditions, such as light, CO_2_, and humidity (Hedrich, 2005; Shimazaki et al., 2007). Previous studies have shown that the regulation of stomatal opening by environmental signals depends on synergistic changes in guard cell turgor (ion flux and sugar), cytoskeletal organization, membrane transport, and gene expression (Hetherington, 2001; Ng et al., 2001; Roux and Leonhardt, 2018; Plackett et al., 2021). Various factors of light conditions, including light intensity, photoperiod, and light quality, can cause a variety of physiological responses in plants (Kinoshita and Shimazaki, 1999; Frechilla et al., 2000; Czekus et al., 2020; Yamori et al., 2020; Appolloni et al., 2021; Trojak et al., 2021). Already in the 1970s, the research showed that various wavelengths of light had differing effects on photosynthesis on a quantum yield basis (Balegh and Biddulph, 1970; Mccree, 1971; Nobel et al., 1975; Katsumi, 1976; Evans, 1987). In particular, red light and blue light, as the most efficient spectra of photosynthesis, are widely used in recently developed plant production plants with artificial lighting (Goto, 2012). Nevertheless, the vital role of green light affecting plant physiological activities was gradually proved in the previous studies (Frechilla et al., 2000; Talbott et al., 2002; McCoshum and Kiss, 2011). While red light and blue light are efficiently filtered from incident light by photosynthetic pigments, green light is transmitted through the canopy that has higher transmittance (Dou et al., 2019; Schenkels et al., 2020). Moreover, green radiation penetrates deeper in the leaf profile than blue or red radiation, scatters between cellular components within the leaf, and drives photosynthesis through abundant lower chloroplasts (Terashima et al., 2009). Furthermore, Frechilla et al. (2000) also documented that green light was able to reverse the previous blue light-induced stomatal opening response. Previous reports have shown that green light has a regulating effect on stomata (Frechilla et al., 2000; Talbott et al., 2002), could promote the growth of plants (Kaiser et al., 2019; Meng et al., 2020; Schenkels et al., 2020; Kusuma et al., 2021; Trojak et al., 2021), partial remediated photosynthetic capacity (Claypool and Lieth, 2021), and increased the nutritional quality of plants (Bian et al., 2018a; Li et al., 2021). Moreover, the inclusion of green light in the growth spectrum reduced stomatal conductance (g_s_) and transpiration and altered stomatal traits, thus improving water-use efficiency and improving drought tolerance (Bian et al., 2019; Trojak et al., 2021). According to Bian et al. (2019), under short-term drought stress, green light significantly decreased g_s_ and increased the intrinsic water-use efficiency (WUE) and instantaneous water-use efficiency (iWUE) of tomato, increased mesophyll conductance, and remained high photosynthetic capacity, which resulted in enhanced drought tolerance. Additionally, in 2021 (Bian et al., 2021), their team further used transcriptomics to analyze the effects of green light on tomato growth and drought stress tolerance, which confirmed that green light has a positive function in alleviating the detrimental effects of drought stress on plant growth and photosynthetic capacity *via* stomatal aperture regulation, and suggested that abscisic acid (ABA)-dependent pathway is involved in green light-induced drought tolerance through stomatal regulation. However, whether there are other ways that green light regulates plant growth is unknown. Moreover, our understanding of the role of light in regulating stomatal processes is still limited. Therefore, the aims of our experiment were to study the effect of green light on the growth and development of cucumber seedlings under drought stress by regulating the amount of red light and blue base light relative to green light and to explore the mechanism of green light partial replacement of red light and blue light in improving plant drought tolerance, so as to provide a theoretical basis for green light to regulate the drought tolerance of cucumber.

## Materials and Methods

### Plant Material and Growth Condition

Cucumber seeds (*Cucumis sativus* L. cv. “Xinchun No. 4”), surface sterilized with liquor potassii permanganatis (0.03%) for 10 min, were soaked in distilled water for 8 h and then grown in dark for 48 h. These germinated seeds were sown in plugs (hole number 50) containing substrate (Gansu Lvneng Ruiqi Agricultural Science and Technology Co. Ltd. Lanzhou, China) and grown under white LED light (Ruiqi, T5, Chain) in an environmentally controlled growth chamber. The light intensity was 150 μmol·m^−2^·s^−1^, day/night temperature is 25/20°C, air humidity is 50%, and photoperiod was 12 h. At about 20 days after germination, healthy and similarly sized seedlings with two true leaves were transplanted into a feeding block (10 × 10 × 8 cm^3^) filled with substrate and watered until they reached full water-holding capacity.

### Drought Treatment and Light Conditions

Then,1 day after transplantation, seedlings were transplanted in a plant culture rack with an artificial light source (PRAL), in which the day/night temperature was 25/20°C, relative humidity was 40–50% and started drought-stressed, which was applied by withholding watering (Xu et al., 2021) at the same time. The relative soil water content corresponding to different drought degrees was added in the Supplementary Materials, which are presented in Supplementary Table 1.

The light treatment was delivered by red, blue, and green light-emitting diode (LED) light. The LED panels (Datang New Energy Technology Co., Ltd., Shenzhen, China) were equipped on cultivation shelves. The distance between the LED panels and the culture bed can be changed to keep the photosynthetic photon flux density [(PPFD), μmol m^−2^s^−1^] at the same level. An opaque white plastic reflective film was placed around the LED panels to ensure uniform radiation on the surface of the culture beds and to prevent light pollution from the adjacent treatments. Plants shift positions every day and plant flats are elevated as needed to ensure uniform PPFD in all light treatments.

**Figure 1:**
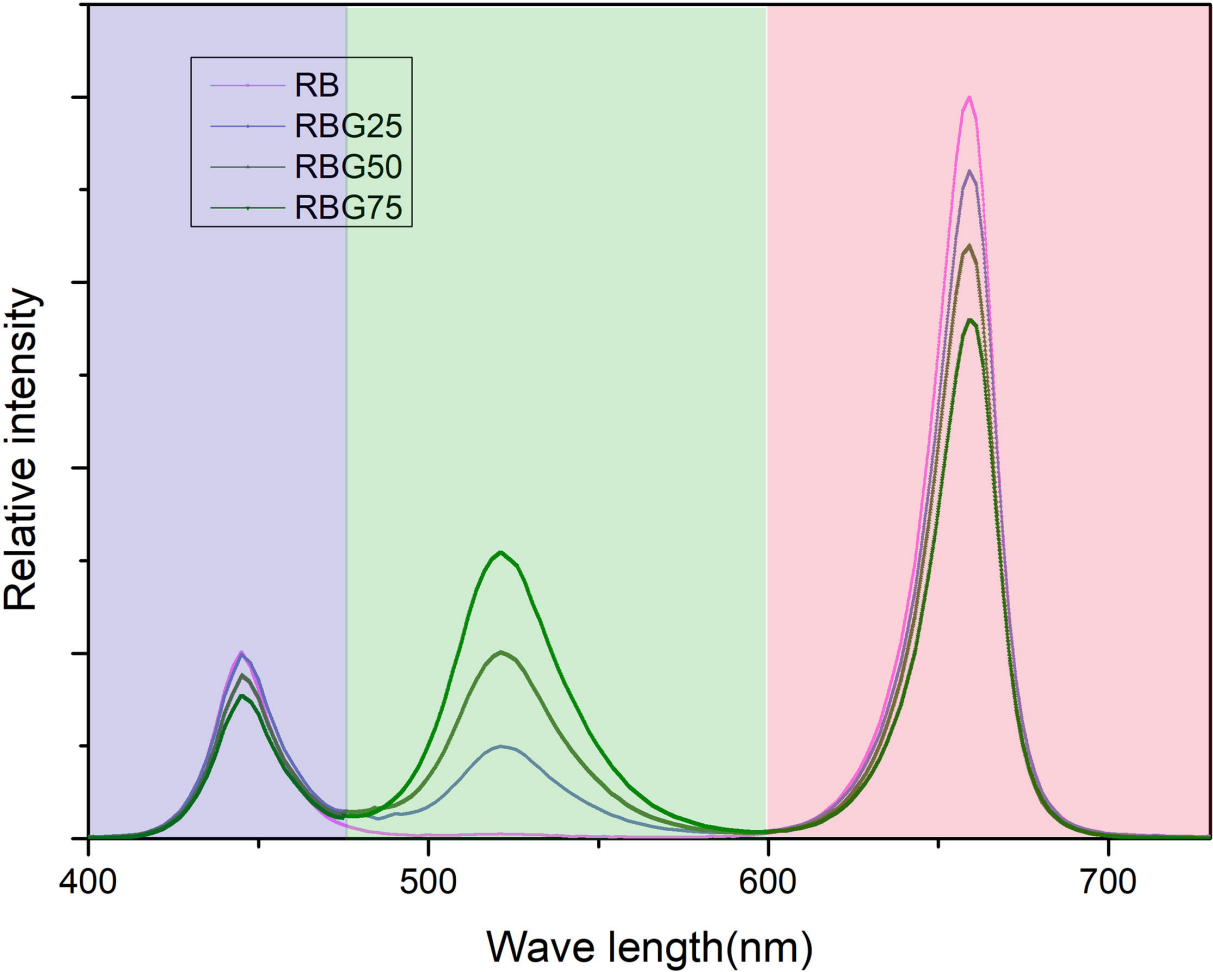
The relative spectral distribution of four LED treatments measured at the top of the canopy. The four LED treatments contained red + blue (RB), red + blue + 25 μmol m^−2^ s^−1^ green (RBG25), red + blue + 50 μmol m^−2^ s^−1^ green (RBG50), red + blue + 75 μmol m^−2^ s^−1^ green (RBG75) LEDs. Light qualities were indicated according to wavelengths: red (peak at 660 nm), blue (peak at 450 nm), and green (peak at 530 nm). The photosynthetic photon flux density (PPFD) of all treatments was maintained at 250 μmol m^−2^ s^−1^. The red to blue (R/B) ratio of all treatments was 4:1.

The PPFD of all treatments was maintained at 250 μmol m^−2^ s^−1^. In addition, the light treatment was delivered by red (peak at 660 nm), blue (peak at 450 nm), and green (peak at 530 nm) LED light. The red-to-blue (R/B) ratio of all treatments was 4:1. The relative amount of green light of each treatment was adjusted to 0 (RB), 25 (RBG25), 50 (RBG50), and 75 (RBG75) μmol m^−2^s^−1^. A total of four different combinations of light treatments together with drought conditions were used in this study. The light spectrum of each treatment is presented in Figure 1. The detailed information of these treatments is summarized in Table 1.

**Table 1:** Total photosynthetic photon flux density (PPFD) and PPFD of red (660 nm), blue (450 nm), and green (530 nm) for each light treatment.

**Treatment**	**PPFD(μmol m^−2^ s^−1^)**		
	**Red**	**Blue**	**Green**
RB	200	50	0
RBG25	180	45	25
RBG50	160	40	50
RBG75	140	35	75

### Determinations of Plant Growth and Morphology

After being treated for 9 days, the second fully expanded leaves from the bottom of the plants were collected and immediately frozen in liquid nitrogen before being stored at −80°C. A total of fifteen plants were randomly selected from each treatment for measuring plant height and stem diameter, and five of them were randomly selected to measure dry weight (DW), fresh weight (FW), leaf surface area, and root morphological indexes. Plant height was measured from the base of the stem to the top of the main stem. The stem diameter was measured from about 0.5 cm above the base of the stem. Leaf surface area and root morphological indexes, including total root length, root surface area, and root volume, were scanned with a root scanner (STD 4800, EPSON, Quebec, QC, Canada) to obtain a digital image, and then, the data were obtained with the aid of the WinRHIZO software (Regent Instruments Inc., Quebec, QC, Canada). A total of fourteen plants, randomly selected from each treatment, were cut into (0.5 cm^2^) leaf disks with a punch, to measure leaf dry mass per unit area (LMA) and leaf moisture status. The LMA was calculated according to the method of Hernández and Kubota (2016). A total of one hundred leaf disks, taken from each sample leaf, were kept in oven first at 105°C for 15 min and then 80°C until constant weight. LMA was calculated as the ratio of leaf area to dry mass.

The shoots and roots of plants were kept in oven first at 105°C for 15 min and then 80°C until constant weight. The FW and DW of shoot and roots were measured by an electronic balance.

### Determination of Leaf Moisture Status

The moisture status of leaves was measured according to the method of Wang (2006). A total of one hundred and 10 leaf disks (0.5 cm^2^) were taken from each sample leaf: fifty of them to measure leaves' total moisture content, fifty of them to measure the contents of free water and bound water, 10 of them to measure leaf water potential. The leaves' total moisture content was calculated as the ratio of the difference value between FW and DW to DW. The contents of free water and bound water were determined by Marlin Chick method (Wang, 2006). Leaf water potential was measured by small flow method (Wang, 2006).

### Determinations of Gas Exchange Parameters and Photosynthetic Pigments

Gas exchange indexes including the net photosynthetic rate (P_n_), stomata conductance (g_s_), and transpiration rate (E) of the second true leaf from bottom of sampled seedlings after treating 3, 6, and 9 days were determined with portable photosynthetic system (CIRAS-2, PP System, the United Kingdom), respectively. A total of three plants were randomly selected from each treatment. The photosynthetic photon flux density (400 μmol m^−2^ s^−1^), ambient CO_2_ concentration (380 μmol mol^−1^), leaf temperature (25°C), and relative humidity (70%) were maintained throughout the measurements. In addition, the WUE and the iWUE of detached leaves were calculated as the ratio of P_n_ vs. E and P_n_ vs. g_s_, respectively, according to Leakey et al. (2019).

After treating for 9 days, the first leaves from the bottom of three randomly selected plants of each treatment were used to measure the photosynthetic pigments. The leaf tissue (0.1 g FW) was soaked in 10 ml 80% (v/v) acetone until the leaf color turned white; afterward, the absorbance of the extract was read at 663, 645, and 440 nm, and pigment (chlorophylls a, b and total carotenoid) contents were calculated according to Lichtenthaler and Wellburn (1983).

### Determination of Electrolyte Leakage and Malondialdehyde (MDA)

The electrolyte leakage was determined as described by Jungklang et al. (2017). Briefly, five leaves' disks with same size (about 0.1 g) punched from the lowest main leaves of three randomly selected plants from each treatment were put into a test tube with 10 ml of distilled water. After soaked for 12 h at room temperature, a conductivity meter was used to measure conductivity, recorded as R_1_. Then, test tubes were boiled for 30 min, and the conductivity was measured, recorded as R_2_. Relative conductivity rate was calculated by (R1/R2) × 100%.

The MDA contents were spectrophotometrically determined using an MDA content detection Kit (BC0020, Solarbio Science & Technology Co., Ltd). Briefly, 1 ml extract was added to 0.1 g leaf tissue for ice bath homogenization. Then, the supernatant was centrifuged at 8,000 g at 4°C for 10 min and extracted, and the drug was added according to the instructions. The mixture was kept warm for 60 min in a water bath at 100°C (covered tightly to prevent water loss) and then cooled in an ice bath at room temperature and centrifuged at 10,000 g for 10 min. The supernatant was removed into a 1-ml glass colorimetric dish. The absorbance monitored at 450, 532, and 600 nm was used to calculate the MDA content against the blank prepared by replacing the sample with an extraction medium.

### Determination of Hydrogen Peroxide (H_2_O_2_) and Superoxide Anion (${\text{O}}_{2}^{-}$) Contents

H_2_O_2_ contents of cucumber leaves were spectrophotometrically determined using a H_2_O_2_ content detection kit (BC3590, Solarbio Science & Technology Co., Ltd). Briefly, 1 ml extract was added to 0.1 g leaf tissue for ice bath homogenization. Then, the supernatant was centrifuged at 8,000 g at 4°C for 10 min and extracted, and the drug was added according to the instructions. The absorbance monitored at 415 nm was used to calculate the H_2_O_2_ content against the blank prepared by replacing the sample with an extraction medium.

The ${\text{O}}_{2}^{-}$ contents of cucumber leaves were spectrophotometrically determined using ${\text{O}}_{2}^{-}$ content detection kit (BC1290, Solarbio Science & Technology Co., Ltd). Briefly, 1 ml extract was added to 0.1 g leaf tissue for ice bath homogenization. Then, the supernatant was centrifuged at 12,000 g at 4°C for 20 min and extracted, and the drug was added according to the instructions. The mixture was centrifuged at 8,000 g at 25°C for 5 min. The absorbance monitored at 415 nm was used to calculate the ${\text{O}}_{2}^{-}$ content. The standard solution was diluted to 0.125, 0.0625, 0.03125, 0.015625, 0.0078125, and 0.0039 μmol/ml, using these standard tubes as the standard curve.

### Observation of Stomata

Fully expanded leaves at a similar position (one leaf per plant, five plants per treatment) were sampled and immediately treated with transparent nail polish to obtain slides of the leaf epidermal fingerprints. The slides were analyzed by microscopy (Eclipse E200, Nikon Inc., Japan) combined with DS-Fi3 camera (1/2 resizing camera mode, active pixels: width 1,440 × height 1,024, maximum frame rate: 30 fps). Stomatal aperture and density were analyzed using particle analysis (http://rsbweb.nih.gov/ij/).

### Determinations of ABA Content

The analysis of ABA content was determined using high-performance liquid chromatography (HPLC) followed the method described by Speirs et al. (2013) with a few modifications. First, at the extraction of the hormone, 0.5 g leaf tissue was quickly ground into a powder with liquid nitrogen, put into 10-ml centrifuge tube, added 5 ml extract (n-propanol: distilled water: hydrochloric acid = 2:1:0.002, V:V), and shook on a shaking table at 100 RPM and 4°C for 30 min. The centrifugal tube was removed, and 2 ml dichloromethane was added and shook again for 30 min. The supernatant was centrifuged at 13,000 RPM at 4°C for 5 min. About 2 ml of the supernatant was absorbed into a lyophilized bottle, placed at −80°C for 14 h, and then freeze-dried into white powder in a freeze dryer (24 h). Then, 200 μl 50% methanol solution was added, in a 0.22-μm organic filter membrane filtration to rediscover the machine detection. The chromatographic analysis using an Agilent 1290-6460 Series Triple Quad liquid chromatography (LC)-mass spectrometer (MS)/MS, equipped with Agilent 1200 series HPLC (Agilent Technologies) using a Phenomenex C18 column (150 mm × 2.1 mm × 8 μm) with a column temperature set at 35°C. Solvents were nanopure water, 10% formic acid aqueous solution, acetonitrile, and methyl alcohol. Elution mode is equal to elution.

### Determinations of γ-Aminobutyric Acid (GABA) Content

The concentration of GABA was assayed using high-performance liquid chromatography (HPLC) according to the method described by Xu et al. (2021) with a few modifications. First, 0.5 g of grinding and mixing sample was accurately weighed, 2 ml of 60% ethanol solution was added, and the vortex was oscillated for 10 min. Ultrasonic extraction at room temperature was 40 min. The supernatant was then centrifuged at 4,000 r/min for 15 min, and all the supernatants were transferred to another centrifuge tube. Extraction was repeated one time, the supernatant was combined two times, nitrogen was blown to the water phase, and finally, the volume was fixed to 4 ml with ultrapure water. Second, for derivation, 0.5 ml of extracted sample solution was taken in the previous step and then 1 ml of 0.5 mol/ L sodium bicarbonate solution with pH9.0 was added, and then 1 ml of DNFB solution was added and vortex mixed for 1 min and then put the mixture in a prepared 60°C thermostatic water bath to react for 60 min (avoid light). After the reaction, when the mixture was cooled to room temperature, phosphate buffer solution with pH 7.0 was added into the mixture to make the volume to 5 ml. After standing for 15 min with shading, 1ml of the reaction solution was filtered with a 0.22μm filter membrane to measure the content of GABA. The chromatographic analysis using a Agilent HPLC (Agilent 1100, USA). This equipment has a DAD detector, Symmetry-C18 column (4.6 × 250 mm, 0.5 μm) with a column temperature set at 40°C, water flows at 1 ml/min, mobile phase containing 1 mol/L sodium acetate solution with pH 5.3, and a mixture of methyl alcohol and water [methyl alcohol: water = 1:1 (V:V)]. Elution mode is equal to elution.

### Total RNA Extraction and Real-Time qRT-PCR

To elucidate the effects of green light on the relative expression levels of GABA synthesis-related and stomatal aperture-related genes, the second true leaf from the bottom of randomly selected nine plants (three plants per sample, three samples per treatment) was collected after treating 9 days from each treatment. Total RNAs were extracted using an RNA simple Total RNA kit (Tiangen Biotech Co. Ltd., Beijing, China) according to the manufacturer's instructions. Complementary DNA (cDNA) was synthesized from RNA (1 μg) using the FastKing gDNA Dispelling RT SuperMix kit (Tiangen Biotech Beijing Co. Ltd., Beijing, China) in 20 μl of reaction mixture containing 2 μl of cDNA sample (100 ng). Quantitative reverse-transcription PCR was performed using SuperReal PreMix Plus kit (SYBR Green) (Tiangen Biotech Co. Ltd., Beijing, China) using a LightCycler® 96 System (Roche Molecular Systems, Inc. Germany). Relative gene expression was evaluated using the 2^−Δ*ΔCt*^ method (Livak and Schmittgen, 2001).

The sequences of the selected genes were obtained from the cucumber genome database (www.cucurbitgenomics.org). The primers of genes were designed using Primer Premier 5 software (Premier Biosoft, Palo Alto, CA, USA) and synthesized by Sangon (Sangon Biotech Co. Ltd., Shanghai, China). Actin gene was used as an internal control. The gene-specific primer sequences are listed in Table 2.

**Table 2:** Primers used for gene expression analysis.

**Gene name**	**Primer sequence (5^′^ → 3^′^)**
*CsActin*	F:GCCAGTGGTCGTACAACAGGTATC
	R:AGCAAGGTCGAGACGGAGGATAG
*CsGAD2*	F:TGCTGGAATTGGTTGGGTTATCTGG
	R:AATTGAGGGTGAAAGTGGGTTGGTC
*CsALMT9*	F:CCGCTACTCTGTTTGGGCTATTCTC
	R:AGTGTGCCAATTCCACGGTTCAG

### Statistical Analyses

All the experiments were performed with at least three independent replicates, and results were expressed as mean ± SE. The least significance difference (LSD) test was performed to compare means at a 5% level of probability. Analysis of variance was performed using SPSS 22.0 (SPSS Institute Inc., the United States), and treatment means were compared using the Tukey's test at a 0.05 level of probability. All figures were prepared with OriginPro 8.5.1 SR2 (OriginLab Institute Inc., the United States).

## Results

### Green Light Partially Replaced Red Light and Blue Light Contributes to the Growth of Cucumber Seedlings Under Drought Condition

**Figure 2:**
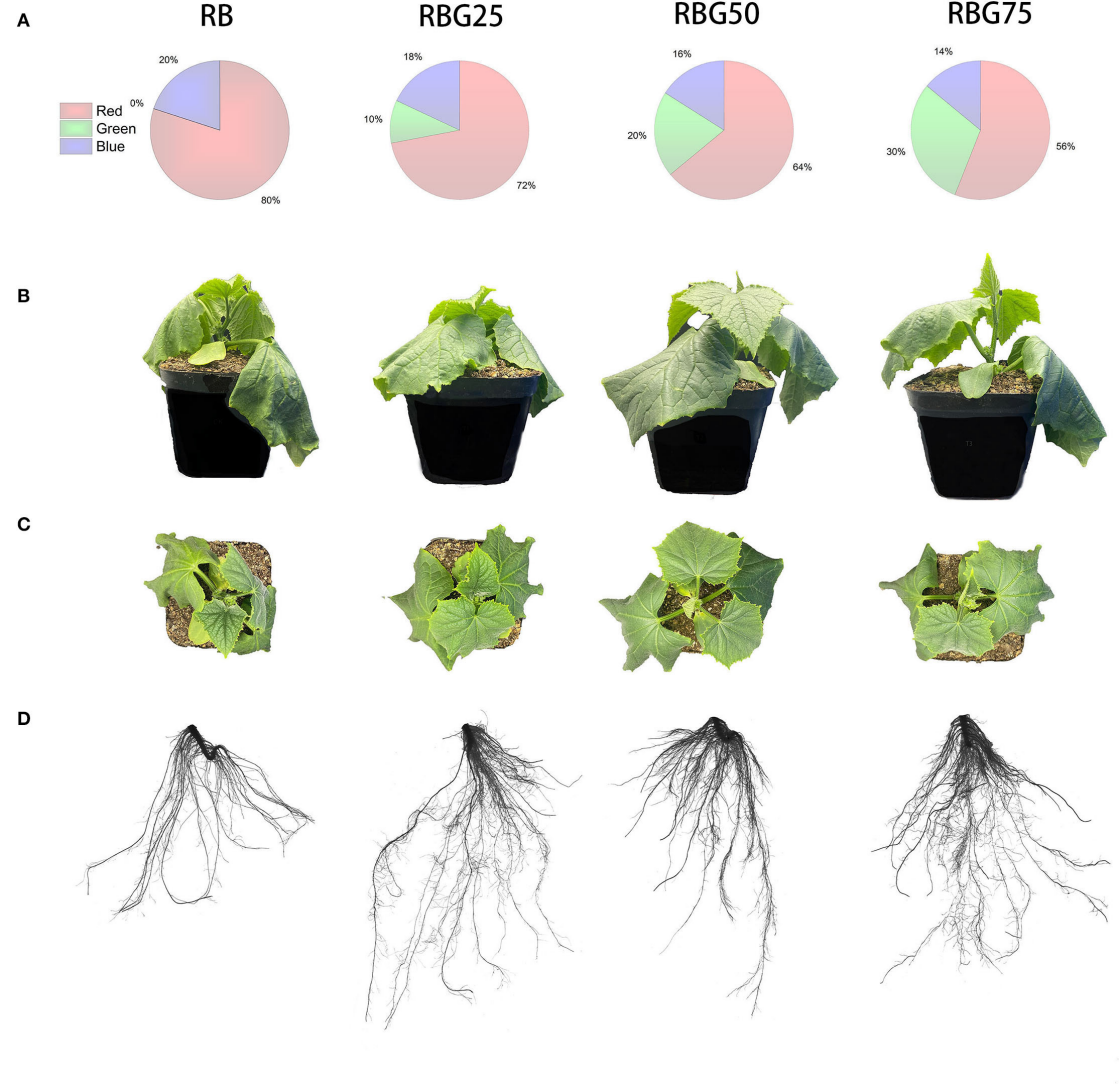
Morphology parameters of cucumber seedlings under different treatments after 9 days drought stress. **(A)** Different spectral conditions include RB, RBG25, RBG50, and RBG75. The different colors represent different light qualities in disc figures. **(B)** Front view of cucumber plants. **(C)** Top view of cucumber plants. **(D)** Root scanned images of cucumber plants. RB, RBG25, RBG50 and RBG75 are the different spectral conditions, respectively. The PPFD for all the treatments was 250 μmol m^−2^ s^−1^. Representative plants were chosen based on plant dry weight and height closest to the average of the treatment. This image is a composite to allow for visual comparison between treatments.

The 9th day's growing conditions were analyzed under four treatments (RB, RBG25, RBG50, and RBG75). The plant growth except for the root FW was significantly affected by light spectral composition under drought conditions (Table 3; Figure 2). As shown in Table 3, compared with the control (RB), green light partial replacement of red light and blue light improved the plant growth under drought conditions, as shown by the higher shoot FW, DW of shoot and root, plant height, stem diameter, leaf area, and LMA.

**Table 3:** The morphological indexes of cucumber seedlings under different light spectral conditions after 9 days of drought stress.

**Treatment**	**Fresh weight (g)**		**Dry weight (g)**		**Plant height (mm)**	**Stem diameter (mm)**	**Leaf area (cm^**2**^)**	**LMA (g m^**−2**^)**
	**Shoot**	**Root**	**Shoot**	**Root**				
RB	5.94 ± 0.12 b	1.05 ± 0.04 a	0.78 + 0.01 b	0.062 ± 0.003 b	54.34 + 0.46 c	4.29 + 0.05 c	427.53 ± 3.36 c	27.71 ± 0.49 b
RBG25	6.26 ± 0.09 ab	1.13 ± 0.04 a	0.82 + 0.01 a	0.093 ± 0.003 ab	56.72 + 0.55 b	4.51 + 0.06 b	457.82 ± 6.7 a	30.85 ± 0.74 a
RBG50	6.57 ± 0.14 a	1.58 ± 0.23 a	0.84 + 0.01 a	0.103 ± 0.015 a	57.72 + 0.68 b	4.72 + 0.07 a	445.05 ± 4.92 ab	31.15 ± 0.99 a
RBG75	6.48 ± 0.19 a	1.55 ± 0.24 a	0.83 + 0.02 a	0.096 ± 0.014 ab	60.97 + 0.86 a	4.33 + 0.04 c	439.23 ± 4.15 bc	29.07 ± 0.71 ab

*The data are presented as Mean ± SEs (n = 3). The different letters in each column indicated significant differences (p < 0.05) among treatments*.

The shoot FW was significantly higher under RBG50 and RBG75 than RB, with respective growth rates of 10.61 and 9.09%, but there were no significant differences between RBG25 and RB. The shoot DW under all the green light supplementation treatments was remarkably higher than that under RB. Compared with RB, the increased rate of the shoot DW under RBG25, RBG50, and RBG75 was 1, 2, and 3%, respectively. The root DW under RBG50 was remarkably higher than that under RB, which was 66.13% higher than RB, but there were no significant differences with other treatments.

The plant height, under all the green light supplementation treatments, was remarkably higher than that under RB, especially RBG75. Compared with RB, the increased rate of the plant height under RBG25, RBG50, and RBG75 was 4.38, 6.22, and 12.20%, respectively. The stem diameter was highest under RBG50, followed by RBG25. Compared with RB, the increased rate of the stem diameter under RBG25 and RBG50 was 5.13 and 10.12%, respectively. But there were no significant differences in stem diameter between RBG25 and RB.

The leaf area was highest under RBG25, followed by RBG50, lowest under RB. In comparison with RB, the leaf area of RBG25 and RBG50 increased by 7.08 and 4.10%, respectively. The LMA under RBG25 and RBG50 was significantly higher than that under RB. In comparison with RB, the leaf area of RBG25 and RBG50 increased by 7.08 and 4.10%, respectively.

These results indicated that green light partial replacement of red light and blue light could promote the growth of cucumber seedlings to some extent under drought conditions.

### Green Light Partially Replaced Red Light and Blue Light Contributes to the Root Growth of Cucumber Seedlings Under Drought Stress

The total root length, average root diameter, total root volume, and root tip number of cucumber seedlings at 9th day under different light treatments are shown in Table 4 and Figure 2. Under drought conditions, compared with the control (RB), the total root volume under RBG25, RBG50, and RBG75 increased by 33.33, 93.33, and 111.11%, respectively. The average root diameter under RBG50 and RBG75 was significantly higher than RB, which increased by 34.67 and 26.67%, respectively. The root tip number under RBG25 and RBG50 was significantly higher than RB, which increased by 106.67 and 78.35%, respectively. But there were no significant differences in total root length between different treatments.

**Table 4:** Effects of different light spectral conditions on total root length, average root diameter, total root volume, and root tip number of cucumber seedlings after 9 days of drought stress.

**Treatment**	**Length (cm)**	**AvgDiam (mm)**	**RootVolume (cm^**3**^)**	**Tips**
RB	645.02 ± 41.53 a	0.300 ± 0.018 b	0.45 ± 0.04 c	2,283.8 ± 347.37 b
RBG25	830.90 ± 101.95 a	0.310 ± 0.016 b	0.60 ± 0.02 b	4,720.0 ± 593.66 a
RBG50	680.17 ± 33.32 a	0.404 ± 0.022 a	0.87 ± 0.07 a	4,073.2 ± 375.85 a
RBG75	880.22 ± 116.18 a	0.380 ± 0.024 a	0.95 ± 0.02 a	3,716.2 ± 586.42 ab

*The data are presented as Mean ± SEs (n = 5). The different letters in each column indicated significant differences (p < 0.05) among treatments*.

### Green Light Partially Replaced Red Light and Blue Light Improves the Water Status of Cucumber Seedlings Under Drought Stress

**Figure 3:**
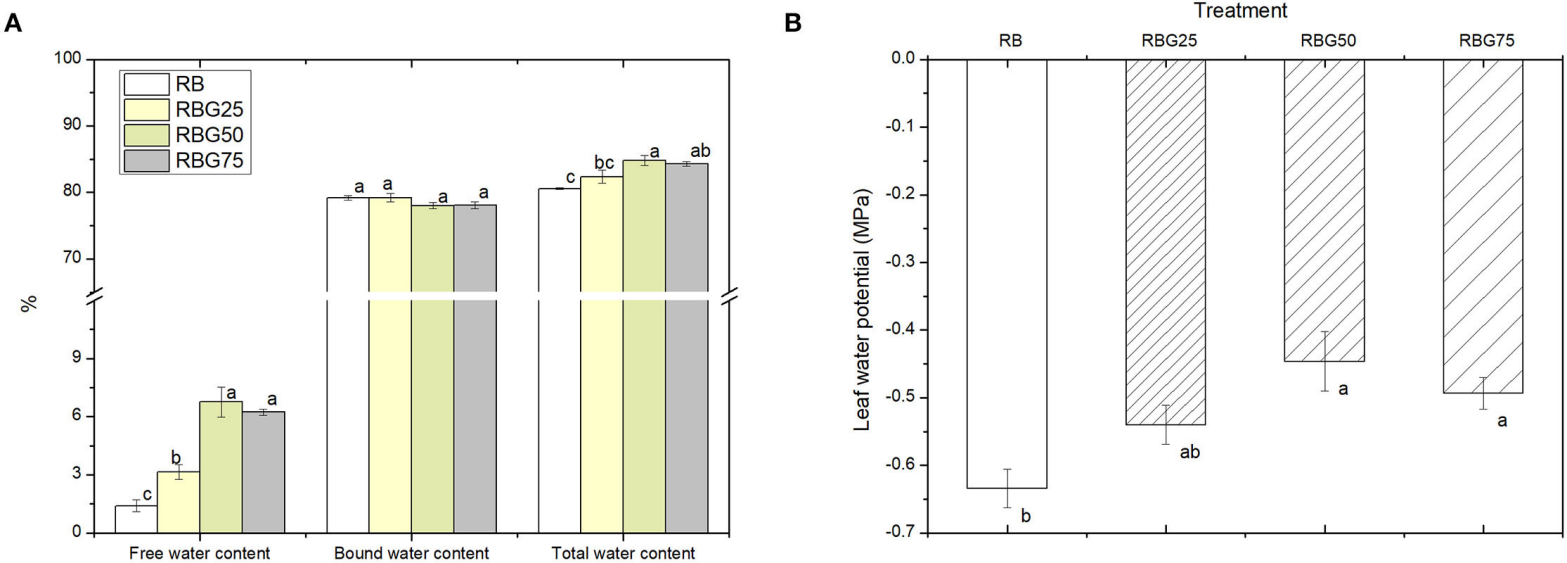
Effect of different spectral conditions on moisture status of cucumber seedlings leaves after 9 days drought stress. **(A)** Free water, bound water and total water content of plant leaves. **(B)** Leaves water potential of plant leaves. RB, RBG25, RBG50 and RBG75 are the different spectral conditions, respectively. The PPFD for all the treatments was 250μmol m^−2^ s^−1^. The data presented were the means ± SE (*n* = 3). Different lowercase letters on the bar chart indicated significant differences among treatments according to Tukey's test (*p* < 0.05).

Under drought stress, compared with RB, RBG50 and RBG75 treatments significantly increased the total water content of leaves (Figure 3A). Free water content, under all green light treatments, was significantly higher than the RB, particularly the RBG50 and RBG75. But there were no significant differences in bound water content between treatments.

The water potential was increased as the green light replaces the part of red light and blue light compared with RB (Figure 3B). In particular, the water potential under RBG50 and RBG75 was significantly higher than RB.

These results showed that green light partial replacement of red light and blue light increased total water content, especially free water content, improved leaf water status, and alleviated water loss in plants caused by drought stress.

### Green Light Partially Replaced Red Light and Blue Light Improves WUE by Decreasing g_s_ and E While Maintaining Higher P_n_

**Figure 4:**
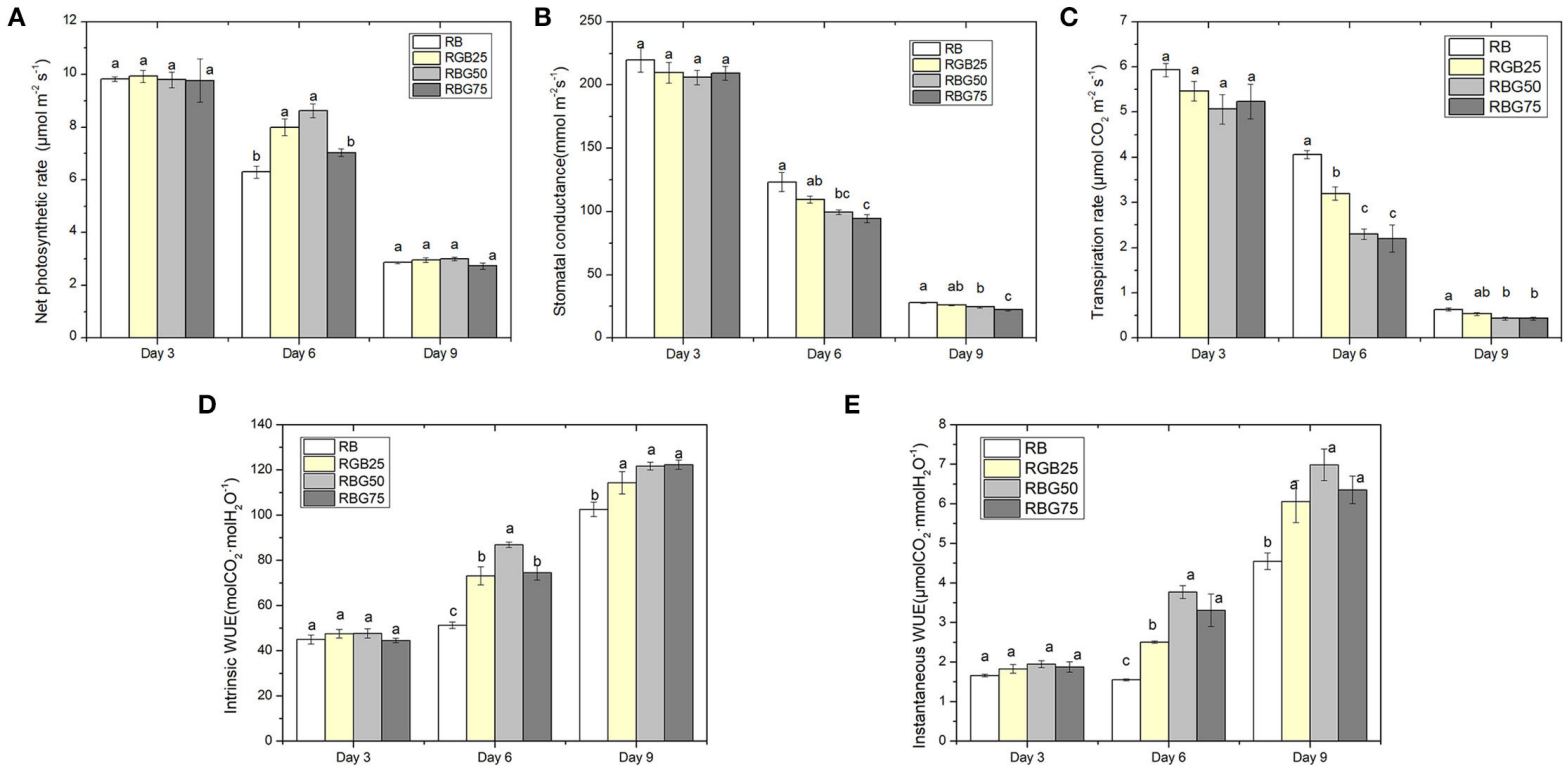
Effect of different spectral conditions on gas exchange parameters of fully expanded cucumber leaves under short-term drought stress. **(A)** Net photosynthetic rate (Pn), **(B)** Stomata conductance (gs), **(C)** Transpiration rate (Tr), **(D)** Lintrinsic water use efficiency (WUE) and **(E)** Instantaneous water use efficiency (iWUE) of cucumber leaves at 3 d, 6 d and 9 d. RB, RBG25, RBG50 and RBG75 are the different spectral conditions, respectively. The PPFD for all the treatments was 250μmol m^−2^ s^−1^. The data presented were the means ± SE (*n* = 3). Different lowercase letters on the bar chart indicated significant differences among treatments according to Tukey's test (*p* < 0.05).

P_n_, g_s_, and E were markedly decreased with processing time (Figures 4A–C). After 6 days of drought treatment, the P_n_ was significantly affected by light spectra. Appropriate green light mitigated the adverse effects of drought on photosynthesis, maintaining photosynthesis at a higher level. However, with the increase in green light content, P_n_ showed a trend of first increasing and then decreasing (Figure 4A).

The influence of light spectra on the change trend of g_s_ is similar to that of E. The green light reduced both g_s_ and E on the 6th and 9th day (Figures 4B,C). Compared with RB, RBG50 and RBG75 significantly decreased g_s_. However, the effect of RBG25 on g_s_ is not significant. The lowest values of g_s_ were detected under RBG75, followed by RBG50 and then RBG25 and RB, at days 6 and 9 (Figure 4B). The E under RBG25, RBG50, and RBG75 were significantly lower than RB at day 6. Moreover, the E under RBG50 and EBG75 were significantly lower than RBG25. The lowest values of E were detected under RBG75 and RBG50, followed RBG25, and then RB, at days 6 and 9 (Figure 4C).

Drought stress led to the increase of WUE and iWUE (Figures 4D,E). Due to the effect of green light, the WUE and iWUE of leaves were significantly enhanced at days 6 and 9. After 6 days of drought treatment, the highest value of WUE was detected under RBG50, followed by RBG25 and RBG75, and then RB (Figure 4D). However, the highest value of iWUE was detected under RBG50 and RBG75, followed by RBG25, and then RB (Figure 4E). The WUE and iWUE between RBG25, RBG50, and RBG75 have no significant differences, which were all apparently higher than RB at day 9.

**Figure 5:**
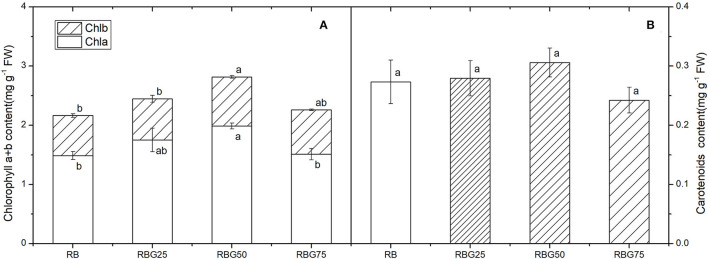
Effect of different spectral conditions on chlorophyll content of cucumber leaves after 9 days drought stress. **(A)** Chlorophyll a and chlorophyll b content of cucumber leaves. **(B)** Carotenoids content of cucumber leaves. RB, RBG25, RBG50 and RBG75 are the different spectral conditions, respectively. The PPFD for all the treatments was 250 μmol m^−2^ s^−1^. The data presented were the means ± SE (*n* = 3). Different lowercase letters on the bar chart indicated significant differences among treatments according to Tukey's test (*p* < 0.05).

Compared with RB, RBG50 significantly increased the content of chlorophylls a and b (Figure 5A). However, there were no significant differences in carotenoids among treatments (Figure 5B).

### Green Light Partially Replaced Red Light and Blue Light Attenuates Lipid Peroxidation of Cucumber Seedlings Caused by Drought Stress

**Figure 6:**
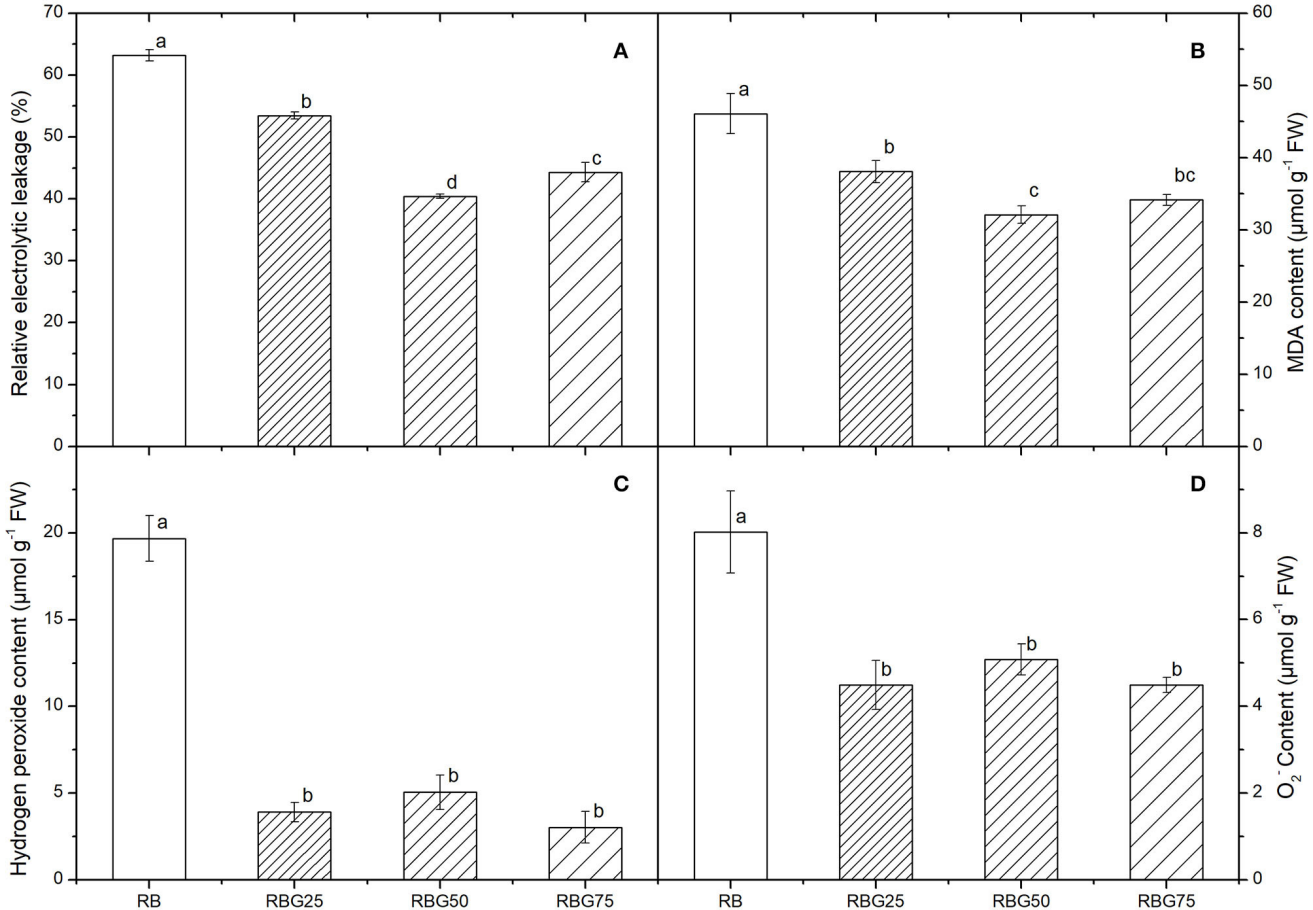
Effect of different spectral conditions on electrolyte leakage, malondialdehyde, hydrogen peroxide and superoxide anion contents of cucumber seeding after 9 days of drought stress. **(A)** The electrolyte leakage. **(B)** Malondialdehyde (MDA) contents. **(C)** Hydrogen peroxide (H2O2) contents. **(D)** Superoxide anion (O^2−^) contents. RB, RBG25, RBG50 and RBG75 are the different light spectral conditions, respectively. The PPFD for all the treatments was 250 μmol m^−2^ s^−1^. The data presented were the means ± SE (*n* = 3). Different lowercase letters on the bar chart indicated significant differences among treatments according to Tukey's test (*p* < 0.05).

After 9 days of drought treatment, the relative electrolytic leakage and MDA content were significantly affected by light spectra (Figures 6A,B). Green light substituting proportion of blue light and red light markedly decreased the relative electrolytic leakage and MDA content compared with RB. The lowest relative electrolyte leakage was observed in RBG50, followed by RBG75, and then RBG25, and the highest was observed in RB (Figure 6A). The effect of light spectrum on MDA content is similar to that of relative electrolyte leakage (Figure 6B).

The contents of ${\text{O}}_{2}^{-}$ and H_2_O_2_ vigorously decreased as the intensity of green light was added to the spectrum under drought (Figures 6C,D). The ${\text{O}}_{2}^{-}$ and H_2_O_2_ contents of RBG25, RBG50, and RBG75 were significantly reduced compared with RB after 9 days of drought treatment (Figures 6C,D).

These results indicated that green light had a positive function in enhancing the plant drought tolerance by alleviating damage caused by drought stress.

### Green Light Partially Replaced Red Light and Blue Light Affects Stomata Morphology and Increases GABA and ABA Contents of Cucumber Seedlings Under Drought Stress

**Figure 7:**
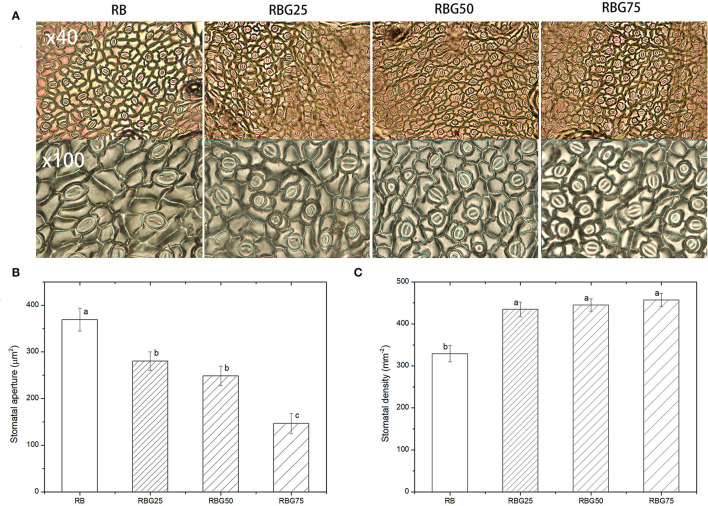
Effect of different spectral conditions on stomatal characteristics of cucumber leaves after 9 days drought stress. **(A)** Images of stomatal. The photos at the top (× 40) are 40 times magnified replica of the stomata. The photos at the bottom (× 100) are 100 times enlarged replica of the stomata. **(B)** Stomatal aperture. **(C)** Stomatal density. RB, RBG25, RBG50 and RBG75 are the different spectral conditions, respectively. The data presented were the means ± SE (*n* = 30). Different lowercase letters on the bar chart indicated significant differences among treatments according to Tukey's test (*p* < 0.05).

**Figure 8:**
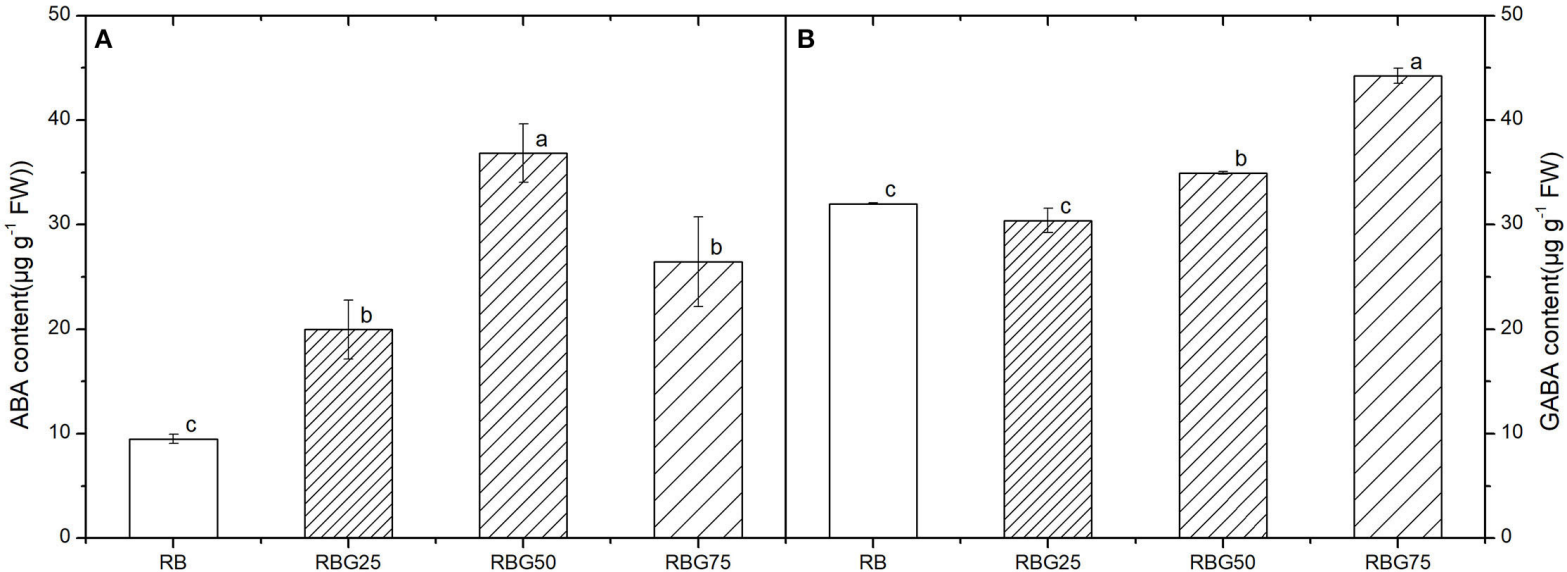
Effect of different spectral conditions on contents of abscisic acid (ABA) and s-aminobutyric acid (GABA) in cucumber seeding leaves after 9 days drought stress. **(A)** Abscisic acid (ABA) content of cucumber leaves. **(B)** γ-aminobutyric acid (GABA) content of cucumber leaves. RB, RBG25, RBG50 and RBG75 are the different spectral conditions, respectively. The PPFD for all the treatments was 250μmol m^−2^ s^−1^. The data presented were the means ± SE (*n* = 3). Different lowercase letters on the bar chart indicated significant differences among treatments according to Tukey's test (*p* < 0.05).

After 9 days of drought treatment, the stomatal morphology, stomatal aperture, density (Figure 7), and GABA and ABA contents (Figure 8) were significantly affected by light spectra. The stomatal development was significantly affected due to the addition of green light. Green light substituting proportion of blue light and red light-induced stomatal closure increased stomatal density, whereas decreased stomatal aperture area (Figure 7). The stomatal aperture under RBG25, RBG50, and RBG75 was significantly smaller than RB, which decreased by 24.08, 32.71, and 60.23%, respectively (Figure 7B). The stomata under RBG25, RBG50, and RBG75 were denser than RB, which increased by 31.98, 35.16, and 38.81%, respectively (Figure 7C). Moreover, adding green light significantly increased GABA and ABA contents (Figures 8A,B), compared with RB. The ABA content was the highest under RBG50, followed by RBG25 and RBG75, whereas the lowest value was observed under RB (Figure 8A). The GABA content was increased with the proportion of green light in the spectrum (Figure 8B).

### Green Light Partially Replaced Red Light and Blue Light Upregulates Transcript Levels of Glutamate Decarboxylase 2 (CsGAD2) but Downregulates the Transcript Levels of Aluminum-Activated Malate Transporter 9 (CsALMT9)

**Figure 9:**
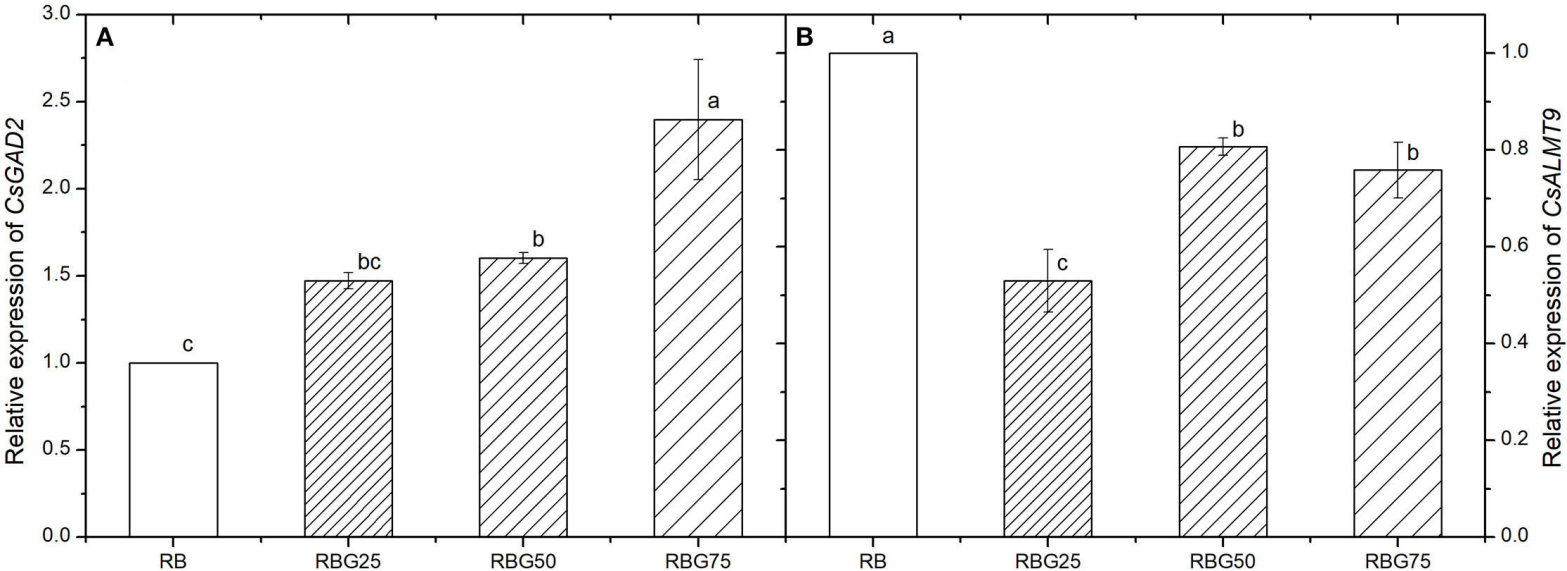
Effect of different spectral conditions on relative expression levels of *CsGAD2* and *CsALMT9* in cucumber seedlings leaves after 9 days of drought stress. **(A)** The relative expression level of *CsGAD2*. **(B)** The relative expression level of *CsALMT9*. RB, RBG25, RBG50 and RBG75 are the different spectral conditions, respectively. The PPFD for all the treatments was 250 μmol m^−2^ s^−1^. The data presented were the means ± SE (*n* = 3). Different lowercase letters on the bar chart indicated significant differences among treatments according to Tukey's test (*p* < 0.05).

The relative expression levels of *CsGAD2* (GABA primarily synthesis gene) and *CsALMT9* (stomatal opening regulation gene) are shown in Figure 9. After 9 days of drought treatment, the transcript levels of *CsGAD2* were upregulated and the transcript levels of *CsALMT9* were downregulated by green light partial replacement of red light and blue light (Figures 9A,B).

## Discussion

In this study, we have demonstrated the positive effects of green light on improving the drought tolerance of cucumber seedlings. For plants, the inhibition of photosynthesis is one of the most important physiological responses to drought stress, which causes the decrease in crop yield. In our present work, green light partial replacement of red light and blue light alleviated the negative effects of drought on the growth of cucumber seedlings (Figures 2, 3; Tables 3, 4), which suggested that the application of green light would obtain higher P_n_ of drought-treated plants (Figure 4A). The above results were consistent with the study by Bian et al. (2018a,b, 2019), who found that green light has a positive effect on maintaining the relatively high photosynthetic capability of plants under abiotic stress. The decrease of photosynthesis under drought conditions might be caused by the stomatal closure and CO_2_ carboxylation efficiency (non-stomatal factor) (Flexas et al., 2008). In our study, green light partially replaced red light and blue light increased ABA and GABA contents, induced stomata to close, and decreased g_s_ and E to reduce water loss, which can enhance the drought tolerance of cucumber seedlings, thus increasing photosynthetic efficiency.

Under drought stress, plants have naturally evolved several different strategies of drought tolerance, such as drought tolerance (the ability of the plant to overcome low tissue water potential) and drought avoidance (the reduction in water loss through stomata and improve water-use efficiency) (Chaves et al., 2003; Hsieh et al., 2010; Kim et al., 2012). In our present work, the significantly high free water content after green light partial replacement of red light and blue light (Figure 3A) might be explained by the reduction of water loss in plant leaves (Figure 3A) with the result that the addition of green light-induced stomatal closure increased stomatal density, whereas stomatal aperture area decreased (Figures 4B, 7). Smaller stomata open and close more quickly, and their general association with high density (Figure 7) provides the ability for a rapid regulation of leaf stomatal conductance, this appoint was supported by the study of Li et al. (2021).

Also, the significantly high leaf water potential and intrinsic and instantaneous WUE after the addition of green light were observed under drought condition (Figures 3B, 4D,E). Together with the low lipid peroxidation and ${\text{O}}_{2}^{-}$ content under green light condition (Figure 6), the above results demonstrated that green light partial replacement of red light and blue light has a positive effect on improving the drought tolerance of cucumber seedling by enhancing water-use efficiency *via* regulating stomatal and decreasing injury induced by drought stress. This appoint was also supported by the study of Bian et al. (2019), who found that green light-induced stomatal responses are the key factor for improving tomato drought tolerance.

Stomatal conductance was mainly affected by stomatal density and stomatal aperture (Dimitrios et al., 2015). Stomatal aperture is controlled by guard cell turgidity, which responds to changes in atmospheric CO_2_ concentration, light, atmospheric relative humidity, and ABA. Moreover, stoma is the gateway for CO_2_ uptake and water loss from plant leaves by transpiration (Osakabe et al., 2014; Assmann and Jegla, 2016; Jezek and Blatt, 2017). Previous researches have shown that blue light can stimulate stomatal opening (Kinoshita and Shimazaki, 1999; Yamauchi et al., 2016), whereas green light reverses blue light-induced stomatal opening (Frechilla et al., 2000; Talbott et al., 2002). This was the reason why stomatal aperture decreased with green light replaced red light and blue light in this study. This was also the main reason that caused lower g_s_ and E under treatments employing green light than that under RB. Moreover, stomatal density is increased by strong light irradiation (Schoch et al., 1980). Due to its high transmittance and reflectivity, green light can penetrate deeper into the canopy (Kasperbauer, 1971; Terashima et al., 2009). Under low light conditions, mature leaves trigger long-distance signal transduction and control stomatal development, leading to a decrease in stomatal density in young leaves (Ehonen et al., 2020). In this experiment, stomatal density increased with the addition of green light. This may be due to the higher transmittance of green light to the plant canopy than red light and blue light. The above result was consistent with the finding that stomatal density increased significantly when blue LEDs were employed (Son and Oh, 2015; Li et al., 2021).

Regulating stomata to reduce water loss is one of the most important strategies to improve water-use efficiency and drought tolerance (Bian et al., 2019, 2021; Xu et al., 2021). A number of the recent studies have shown that green light could alter the stomatal aperture to improve drought tolerance (Frechilla et al., 2000; Talbott et al., 2002; Bian et al., 2019, 2021). However, the mechanisms remain unclear. To understand the effect of green light on drought tolerance, g_s_ and ABA and GABA contents of cucumber seedlings were further studied. Our study showed that compared with the control group, the g_s_ of cucumber seedlings was lower and the water-use efficiency was higher due to the addition of green light, which was consistent with the previous studies (Bian et al., 2019, 2021). The g_s_ and E were lower under green light treatment than those under red light and blue light (Figures 4B,C). These findings may lie in the fact that red light and blue light regulate stomata opening, whereas green light is less efficient in promoting stomatal opening and strong green light suppressed the blue light-dependent stomatal opening (Frechilla et al., 2000; Shimazaki et al., 2007). This study also showed that green light could increase the contents of ABA and GABA. Studies have shown that ABA and GABA can regulate stomata and improve water efficiency (Kim et al., 2012; Xu et al., 2021), which further supports our conclusion. Based on the above results, we hypothesized that green light could increase the contents of GABA and ABA in plants under drought conditions, thus regulating the stomatal aperture and improving water utilization rate. Bian et al. (2021) have used a transcriptome technique to reveal the DEGs involved in ABA synthesis and ABA signal transduction both participated in the green light-induced drought tolerance of tomato plants. However, as a universal stomatal behavior modifier, whether GABA plays a role in regulating drought tolerance of plants under green light has still not clear. To examine the hypothesis that endogenous GABA concentration increases under green light and acts as a signal, we determined GABA level under different treatments and found that GABA accumulation increases under green light when cucumber seedlings exposed to drought. GAD2 plays a prominent role in maintaining the shoot GABA level (Mekonnen et al., 2016). Moreover, Xu et al. (2021) have revealed that ALMT9 is a GABA target that regulates plant water loss, even under nonstressed conditions, through the modulation of ALMT9 activity. Xu et al. (2021) have studied the regulation of GABA on stomatal opening in ALMT9 mutants. The results show that ALMT9 is an important part of GABA signal transduction in guard cells and relies on GABA signal to regulate water-use efficiency and drought recovery ability. GABA reduces the rate or degree of stomatal opening by targeting and inhibiting ALMT9 activity. Therefore, we further investigated the relative expression levels of *CsGAD2*, which is GABA primarily synthesis gene, and *CsALMT9*, which is the stomatal opening regulation gene. The results showed that green light upregulated the relative expression level of *CsGAD2* gene and downregulated the relative expression level of *CsALMT9*, which is consistent with our hypothesis.

**Figure 10:**
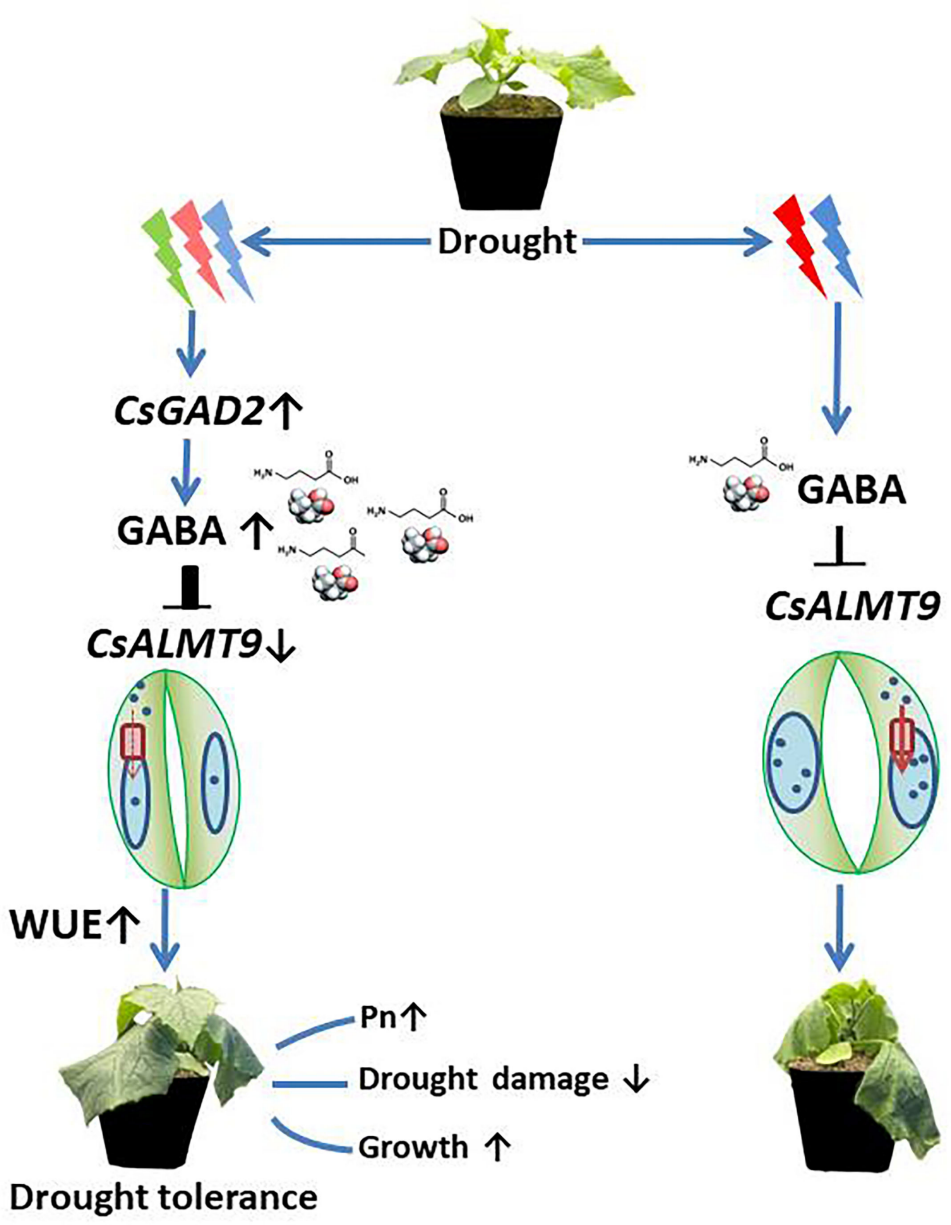
Proposed model of the regulation pathway of green light on plant drought resistance. Green light stimulates the up-regulation of *CsGAD2*, thus promoting the synthesis of GABA, and regulates stomatal opening and water use efficiency drought resistance through negative regulation of the expression level of *CsALMT9*. Since green light replaces part of red and blue light, compared with red and blue light, green light upregulated the expression of *CsGAD2* gene under drought stress and increased the accumulation of GABA in leaves. GABA negatively regulated the uptake of negative ions mediated by down-regulating the relative expression level of *CsALMT9* into protective cell vacuoles, thus fine-tune stomatal opening, reduced stomatal opening and improved water use efficiency (left). In the absence of green light treatment, due to the relatively low expression level of *CsGAD2* gene, the accumulation of GABA in leaves was reduced and ALMT9 was misadjusted, thus maximizing the uptake and accumulation of anions in protective cell vacuoles. Compared with plants treated with green light under red and blue light conditions, the stomata of plant was open widely, resulting in greater water loss and lower water use efficiency (right).

The data in this study have unveiled the regulatory pathway of green light on drought tolerance of cucumber seedlings, which can be summarized by the simplified models presented in Figure 10. We proposed that green light stimulated upregulation of *CsGAD2*, which promotes the synthesis of GABA and then modulates stomatal opening, WUE, and drought-resistant through negative regulation of *CsALMT9* (Figure 10). Further studies at physiological and molecular levels should be conducted to provide more precise insight into the mechanisms of green light-induced drought tolerance. Successful identification of these key genes would give a great opportunity for breeding new varieties with high resource use efficiency or/and stress tolerance that is suitable for vertical farming.

## Conclusion

The evidence in this study supported the following arguments: 1) green light partial replacement of red light and blue light improved drought tolerance of cucumber seedlings; 2) green light partial replacement of red light and blue light causes a decrease in g_s_ and stomatal closure to enhance water-use efficiency by upregulating *CsGAD2* expression to increase GABA content and then downregulating *CsALMT9* expression to enhance cucumber seedling drought tolerance. Moreover, green light partial replacement of red light and blue light reduced the relative electrolytic leakage and contents of MDA, ${\text{O}}_{2}^{-}$, and H_2_O_2_ to alleviate the damage of drought stress on the cells and cell membranes of cucumber seedlings. In the future, transcriptomic data will contribute to our knowledge of the molecular mechanism by which green light regulates photosynthetic capability, stomatal movement, and water-use efficiency.

## Summary Statement

In this paper, we reported for the first time that GABA is an important signal for cucumber to exhibit better drought tolerance by regulating stomata under green light in partial substitution of red + blue light source.

## Data Availability Statement

The datasets presented in this study can be found in online repositories. The names of the repository/repositories and accession number(s) can be found in the article/Supplementary Material.

## Author Contributions

YM, JY, and LH conceived the original research plan and designed the experiment. YM and LH performed the experiments, analyzed the data, reviewed, and edited the manuscript. YM wrote the manuscript. All authors read and approved the final manuscript.

## Funding

This work was supported by the Education Science and Technology Innovation Project of Gansu Province (GSSYLXM-02), the National Natural Science Foundation of China (32160705), the Special Project of Central Government Guiding Local Science and Technology Development (ZCYD-2021-06), and the Agriculture Research System of China (CARS-23-C-07).

## Conflict of Interest

The authors declare that the research was conducted in the absence of any commercial or financial relationships that could be construed as a potential conflict of interest.

## Publisher's Note

All claims expressed in this article are solely those of the authors and do not necessarily represent those of their affiliated organizations, or those of the publisher, the editors and the reviewers. Any product that may be evaluated in this article, or claim that may be made by its manufacturer, is not guaranteed or endorsed by the publisher.
