# Supplementary material for: Green Light Partial Replacement of Red and Blue Light Improved Drought Tolerance by Regulating Water Use Efficiency in Cucumber Seedlings
Source: Front Plant Sci. 2022 May 31;13:878932. doi: 10.3389/fpls.2022.878932 (PMC9194611; doi:10.3389/fpls.2022.878932)
Supplement: Supplementary file 1 [file Table_1.DOCX]

1. Green light improved drought tolerance of cucumber seedlings by enhancing WUE.
2. Green light alleviated the drought-induced inhibition of photosynthetic capacity.
3. Green light alleviated damage caused by drought stress.
4. Green light induced stomatal closure by increasing GABA and ABA content.
