# Supplementary material for: Green Light Partial Replacement of Red and Blue Light Improved Drought Tolerance by Regulating Water Use Efficiency in Cucumber Seedlings
Source: Front Plant Sci. 2022 May 31;13:878932. doi: 10.3389/fpls.2022.878932 (PMC9194611; doi:10.3389/fpls.2022.878932)
Supplement: Supplementary file 3 [file Table_3.DOCX]

[Supplementary Figure 1](Supplementary%20Figure%201.jpg) |**Substrate water content under different treatments at 0, 3, 6, 9 day.** (A) Gravimetric substrate water content. (B) Relative substrate water content. RB, RBG25, RBG50 and RBG75 are the different spectral conditions, respectively. The data presented were the means ± SE (n = 3). Different lowercase letters on the bar chart indicated significant differences among treatments according to Tukey’s test (p < 0.05).
