# Supplementary material for: Green Light Partial Replacement of Red and Blue Light Improved Drought Tolerance by Regulating Water Use Efficiency in Cucumber Seedlings
Source: Front Plant Sci. 2022 May 31;13:878932. doi: 10.3389/fpls.2022.878932 (PMC9194611; doi:10.3389/fpls.2022.878932)
Supplement: Supplementary file 5 [file Table_5.DOCX]

**Supplementary Table 1|** The relative soil water content corresponding to different drought degrees

| Degree of drought | Relative substrate water content (R) |
| --- | --- |
| Over-wet | R>80% |
| wet | 60% <R≤ 80% |
| moderate stress | 40% <R≤60% |
| Severe drought | 20% <R≤ 40% |
| special drought | R≤20% |
