# Supplementary figures and images for: Green Light Partial Replacement of Red and Blue Light Improved Drought Tolerance by Regulating Water Use Efficiency in Cucumber Seedlings
Source: Front Plant Sci. 2022 May 31;13:878932. doi: 10.3389/fpls.2022.878932 (PMC9194611; doi:10.3389/fpls.2022.878932)

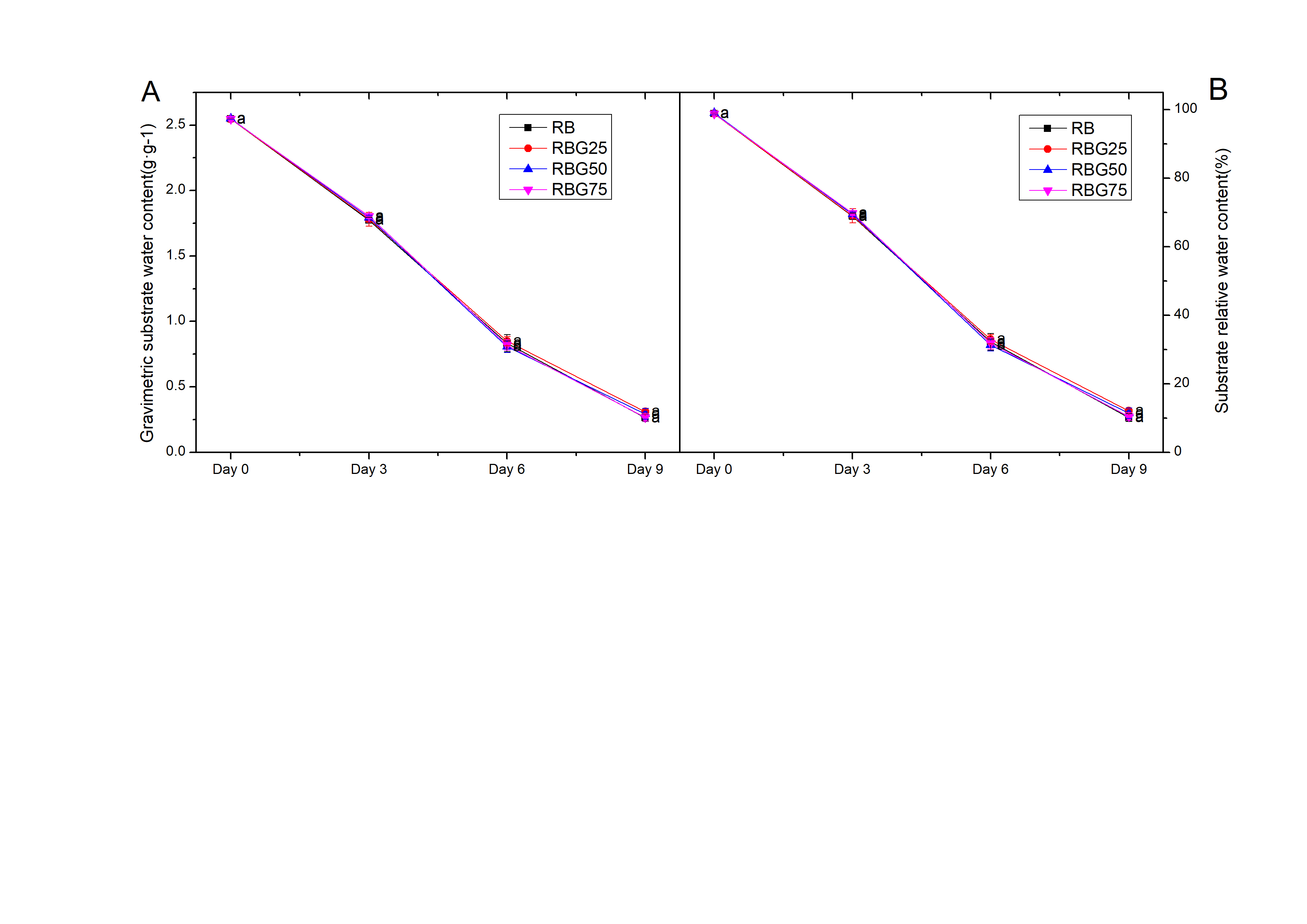

Supplement: Supplementary file 6 [file Image_1.JPEG]
